# Chemotherapy plus Ofatumumab at Standard or Mega dose in relapsed CLL (COSMIC) trial: study protocol for a phase II randomised controlled trial

**DOI:** 10.1186/s13063-016-1581-0

**Published:** 2016-09-20

**Authors:** Dena R. Howard, Talha Munir, Anna Hockaday, Andy C. Rawstron, Laura Collett, Jamie B. Oughton, David Allsup, Adrian Bloor, David Phillips, Peter Hillmen

**Affiliations:** 1 Clinical Trials Research Unit, Leeds Institute of Clinical Trials Research, University of Leeds, Leeds, LS2 9JT UK; 2 St James’s Institute of Oncology, St James’s University Hospital, Leeds, UK; 3 Haematological Malignancy Diagnostic Service, St James’s Institute of Oncology, St James’s University Hospital, Leeds, UK; 4 Department of Haematology, Queens Centre for Oncology and Haematology, Castle Hill Hospital, Hull and East Yorkshire Hospitals NHS Trust, Cottingham, UK; 5 Department of Haematology, The Christie NHS Foundation Trust, University of Manchester, Manchester Academic Health Sciences Centre, Manchester, UK

**Keywords:** CLL, Chronic lymphocytic leukaemia, Ofatumumab, FC, Bendamustine, Phase II trial, MRD, Minimal residual disease, Dose selection, Relapsed population, Randomised clinical trial

## Abstract

**Background:**

Chronic lymphocytic leukaemia (CLL) is the most common adult leukaemia. Combination immunochemotherapy such as fludarabine, cyclophosphamide and rituximab is the standard first line therapy in fit patients, but there is limited evidence regarding the optimal treatment of patients after relapse. Ofatumumab as monotherapy has been proven to be effective in the treatment of relapsed, refractory CLL, and as it is not myelotoxic, it is an ideal drug to combine with chemotherapy. However, the optimal dose of ofatumumab in this setting is not known. The Chemotherapy plus Ofatumumab at Standard or Mega dose in relapsed CLL (COSMIC) trial will assess the efficacy and safety of standard and high (mega) doses of ofatumumab combined with bendamustine or a combination of fludarabine and cyclophosphamide to determine which, if either, schedule should progress to a phase III trial.

**Methods/design:**

COSMIC is a phase II, multi-centre, randomised, open, parallel group trial for patients with relapsed CLL who are not refractory to fludarabine-based chemotherapy. Participants will be randomised to receive either standard dose or mega dose ofatumumab. Both doses will be given in combination with either bendamustine or fludarabine and cyclophosphamide chemotherapy backbone. The primary objective is to assess the proportion of participants achieving a complete remission following therapy with the two treatment arms (mega versus standard), as assessed at 3 months post treatment. The treatment groups will be assessed independently to determine whether the level of response is acceptable in relation to pre-specified criteria. If both treatment groups show an acceptable level of response, selection criteria will be used to determine which to take forward to a confirmatory phase III trial. A key secondary objective is to assess the dynamics of minimal residual disease (MRD) levels in relapsed disease. Eighty-two participants are planned to be recruited from 18 research centres in the UK.

**Discussion:**

Currently there is limited evidence regarding the optimal treatment of patients with relapsed or refractory CLL, and so suitable therapies are urgently needed. The COSMIC trial will identify whether ofatumumab given in combination with chemotherapy is safe and effective in this population, and will identify the optimal doses for further investigation.

**Trial registration:**

ISRCTN51382468. Registered on 21 September 2011.

**Electronic supplementary material:**

The online version of this article (doi:10.1186/s13063-016-1581-0) contains supplementary material, which is available to authorized users.

## Background

### Chronic lymphocytic leukaemia (CLL)

CLL is the most common adult leukaemia, affecting between 1.0 and 5.0 per 100,000 population [1]. The incidence of CLL increases with age, and twice as many men are affected as women. CLL arises as a result of the clonal proliferation of B cells and is diagnosed by the pattern of expression of various cell surface antigens on the CLL cells. Patients most commonly present with lymphocytosis, lymphadenopathy, splenomegaly and systematic symptoms, such as fatigue, weight loss and malaise. The clinical course of CLL is highly variable with a median survival from diagnosis in the region of 7 years, with the survival being significantly worse for patients with more advanced disease.

### Therapy for relapsed CLL

Over the few years prior to designing the Chemotherapy plus Ofatumumab at Standard or Mega dose in relapsed CLL (COSMIC) trial there were substantial improvements in CLL treatment. First line therapy moved away from monotherapy using chlorambucil towards combination immunochemotherapy such as fludarabine, cyclophosphamide and rituximab (FCR), following evidence from large randomised trials [2]. FCR was approved by the National Institute for Health and Care Excellence (NICE) [3]; therefore, every patient deemed fit enough for intensive first line therapy can receive FCR in the UK. This has resulted in an improvement in response rates and survival outcomes.

The treatment of relapsed and refractory CLL is increasingly difficult, as the choice of treatment depends on a variety of factors such as prior therapy, co-morbidities, length of remission and the presence of biological adverse prognostic factors. With the relatively recent introduction of FCR to front line therapy there is no evidence regarding the optimal treatment of patients after relapse following FCR. Therapies are needed for this group of patients, since increasing numbers of patients will be relapsing after FCR in the future.

For patients who have not previously received fludarabine combinations or who have responded to them with a relatively durable remission, fludarabine combinations are an option in this setting. Bendamustine (B) has also become widely used in the treatment of relapsed CLL, where it is felt to be preferable in those patients who are not fit for fludarabine.

Other alternatives for patients with fludarabine-refractory disease or with shorter remissions include monotherapy with antibodies like alemtuzumab and ofatumumab, combination chemotherapy and possibly allogeneic stem cell transplantation.

### Rationale for therapeutic study

Ofatumumab (Arzerra®) is an anti-CD20 antibody which, as a monotherapy, has been demonstrated to be effective in CLL refractory to fludarabine and either unsuitable for, or refractory to, alemtuzumab [4]. More than 50 % of patients with refractory CLL respond to single agent ofatumumab given at high doses (total dose 22.3 g) [4], and this dose is now approved by the Food and Drug Administration (FDA) and European Medicines Agency (EMA) in this setting. The licensed dose of ofatumumab when given as a single agent includes high dose intensity in the first 8 weeks of therapy, with weekly doses of 2000 mg per infusion [4].

The combination of monoclonal antibodies, such as ofatumumab, with chemotherapy has been demonstrated to be effective for a variety of lymphoproliferative disorders, including CLL, with clear synergy between the two treatment modalities. Ofatumumab given at lower doses (total dose 6.3 g) leads to high response rates, when combined with FC, in previously untreated patients with CLL [5]. Therefore, there is a strong rationale to include the combination of ofatumumab and FC at this lower dose within a study in a relapsed setting. The higher approved dose of 22.3 g has been proven to be deliverable and effective, and since the majority of the ofatumumab is given in the first 8 weeks of therapy, and as it is not myelotoxic, combining ofatumumab with the standard FC chemotherapy at this dose is also an appealing proposal. Therefore, the trial is designed to assess standard (6.3 g) dose ofatumumab and mega (22.3 g) dose ofatumumab given in combination with FC.

During recruitment, a major protocol amendment was implemented to allow the chemotherapy backbone to include bendamustine (B) instead of FC, at the discretion of the treating clinician. This was in response to changing clinical practice and in order to open the trial to as many patients as possible without compromising the trial design. Bendamustine has been used in combination with ofatumumab in phase II studies for patients with both previously untreated [6] and relapsed CLL [6, 7]. The results of these studies demonstrated that ofatumumab in combination with bendamustine is an effective and tolerable therapy, providing high response rates, with an acceptable safety profile for patients with relapsed CLL. In addition, the combination of bendamustine with rituximab seems to have similar efficacy in comparison with FC plus rituximab in the relapsed/refractory setting [8], indicating that the change to include bendamustine in COSMIC would not be expected to have a large effect on the response rate.

This trial has therefore been designed as a randomised phase II trial in patients with relapsed CLL, who are not fludarabine refractory, in order in order to assess the response rates of standard (6.3 g) dose ofatumumab and mega (22.3 g) dose ofatumumab, when given in combination with FC/B. The trial is required to determine which, if any, schedule shows adequate efficacy and should be pursued in larger phase III trials.

## Methods/design

### Trial aims and objectives

The aim of the COSMIC trial is to assess the efficacy of standard dose and mega dose ofatumumab in combination with chemotherapy (bendamustine, or fludarabine and cyclophosphamide) in patients with relapsed CLL. The trial will allow the assessment of which schedule, if any, is acceptable in terms of response rate, eradication of detectable minimal residual disease (MRD) and toxicity and should continue to be investigated within a phase III trial setting.

The primary objective is to assess the rate of complete remission (CR or CR(i) [CR with incomplete marrow recovery]) as defined by the International Workshop on CLL (iwCLL) criteria [9], following therapy with Standard Of-FC/B and Mega Of-FC/B.

Secondary objectives are to assess the following: proportion of participants achieving undetectable MRD; overall response rates as defined by iwCLL criteria; progression-free survival; overall survival; time to MRD relapse in MRD-negative participants; dynamics of MRD relapse and its association with disease progression and survival; and safety and toxicity.

### Trial design

This is a phase II, multi-centre, randomised, open, parallel group trial for patients with relapsed CLL who are not refractory to fludarabine-based chemotherapy. Patients with relapsed CLL who require therapy by conventional criteria will be randomised to either standard dose ofatumumab (6.3 g in total) or high dose ofatumumab (22.3 g in total). All participants will also concurrently receive a chemotherapy backbone of either 6 cycles of bendamustine (B) or fludarabine and cyclophosphamide (FC). The choice of backbone therapy regimen must be made prior to randomisation by either the treating clinician or the patient, and will be recorded at the point of randomisation. The trial treatment period is approximately 6 months. A schedule of enrolment, interventions and assessments is provided in Table 1, and a populated Standard Protocol Items: Recommendations for Interventional Trials (SPIRIT) checklist for this manuscript is also provided (Additional file 1).

**Table 1:** Schedule of enrolment, interventions and assessments

	Eligibility screening	Randomisation	Baseline	Start of therapy	After 3 cycles of therapy	At end of therapy	3 months post therapy	12, 18 and 24 months post randomisation	After 24 months post randomisation	Annual follow-up
Enrolment										
Eligibility screening	X									
Informed consent	X									
Medical history	X									
Local chemotherapy decision (FC or B)	X									
Randomisation		X								
Intervention										
Standard Of-FC/B										
Mega Of-FC/B										
Assessments										
CT scan	X						X	X^a^		
Assessment of disease			X		X		X	X		
Peripheral blood for MRD^b^	X				X		X^c^	X^c^	X^c^	
Bone marrow aspirate for MRD^b^	X						X			
Performance status	X		X		X	X	X	X		
Laboratory tests (haematology)	X		X		X	X	X	X		
Laboratory tests (biochemistry)	X		X		X	X				
Body surface area^d^			X		X					
ARs and SAEs		From randomisation until 30 days after the last dose of treatment								
SARs and SUSARs		From randomisation until the end of the trial								
Survival status										X

*Standard Of-FC/B* standard dose ofatumumab + fludarabine and cyclophosphamide/bendamustine, *Mega Of-FC/B* mega dose ofatumumab + fludarabine and cyclophosphamide/bendamustine, *MRD* minimal residual disease, *AR* adverse event, *SAE* serious adverse event, *SAR* serious adverse reaction, *SUSAR* suspected unexpected serious adverse reaction

^a^If appropriate clinically

^b^Tested centrally

^c^Taken on a 3-monthly basis until five consecutive MRD positive results

^**d**^Before each cycle of therapy and dose changed if greater than 10 % change from baseline

### Trial population

Patients who are eligible for the trial must be at least 18 years old, have CLL requiring therapy, have undergone at least one regime of chemotherapy previously and have a life expectancy of at least 12 weeks. The patient must also be capable of giving written consent, be considered fit enough to receive fludarabine-based or bendamustine chemotherapy and have a World Health Organisation (WHO) performance status (PS) of 0, 1 or 2.

Patients with any of the following characteristics are excluded from the trial: refractoriness to the planned chemotherapy backbone (FC/B); deletion of chromosome 17p on fluorescence in situ hybridisation (FISH); previous treatment with ofatumumab either alone or in combination with chemotherapy; previous toxicity to the planned chemotherapy backbone (FC/B); active infection; other severe, concurrent diseases or mental disorders that could interfere with their ability to participate in the study; creatinine clearance of less than 30 mL/min for fludarabine or less than 10 mL/min for bendamustine; pregnant or lactating women, or women/men who are capable of conceiving children and who are unwilling to use appropriate medically approved contraception during and for 12 months after receiving treatment; current active hepatic or biliary disease (with the exception of patients with Gilbert's syndrome, asymptomatic gallstones, liver metastases or stable chronic liver disease per investigator assessment); treatment with any known non-marketed drug substance or experimental therapy within 5 terminal half-lives or 4 weeks prior to enrolment, whichever is longer, or currently participating in any other interventional clinical study; other malignancy within 2 years, except completely resected non-melanoma skin cancer or successfully treated in situ carcinoma; prior treatment with anti-CD20 monoclonal antibody or alemtuzumab within 3 months prior to start of therapy; or chronic or current infectious disease requiring systemic antibiotics, antifungal or antiviral treatment such as, but not limited to, chronic renal infection, chronic chest infection with bronchiectasis, tuberculosis and active hepatitis C.

### Sample size

Eighty-two participants will be randomised to either Standard Of-FC/B or Mega Of-FC/B. The A’Hern exact one-stage design [10] is proposed to assess the efficacy of each treatment arm based on complete remission to treatment. Should both dosing schedules be deemed worthy of further investigation, selection criteria, based on observing a clinically significant difference in complete remission rates, will be applied to determine which treatment to take forward to a phase III trial. The sample size of 37 participants per arm has been inflated by 10 % to allow for participants who withdraw or who do not reach the primary endpoint. Participants who are randomised, but do not receive any of the study treatment, will be replaced.

Based on the results from the NCRI CLL201 trial [11], where 15.4 % of participants on the FCM arm achieved a CR or CR(i) after treatment, it was agreed that a complete remission rate of less than this would be considered unacceptable. It is felt that a response rate of 33 % or greater would be deemed clinically effective. With 80 % power and a one-sided type I error rate of 5 % for incorrectly determining a schedule worthy of further consideration when in fact the response rate is unacceptably low, 10 complete remissions would be required from 37 participants (95 % confidence interval (CI) 15.4–43.0 %) in order for either treatment to be deemed acceptable to be investigated in a larger trial. Under this design, a particular arm will be a failure if 9/37 (24.3 %) or fewer patients achieve a complete remission. In the NCRI CLL201 trial, the 95 % CI for the complete remission rate with fludarabine, cyclophosphamide, mitoxantrone and rituximab (FCM-R) was 25.5–61.0 %, so these treatments would only be deemed a failure if they lie below the confidence interval for the response rate achieved in a similar population with FCM-R.

If both schedules’ responses successfully exceed the efficacy boundaries, selection criteria following the methodology of Sargent and Goldberg [12] will be implemented. If there are less than 3 responses (8 %) difference observed between the arms, the trial will be declared statistically ambiguous, and alternative selection criteria will be used to select the schedule for further investigation. Otherwise, the schedule with the better observed response rate will be recommended to be taken forward. With 37 participants per arm, and assuming that the correct arm would be selected 50 % of the time by chance if the outcome is statistically ambiguous, selection probabilities are estimated based on an assumed complete remission rate of 27 % in the arm with the poorest response rate. If there is an observed difference of 3 responses, there is 72 % probability of correctly identifying the schedule if the true difference between the two schedules is 8 % (3 responses), 80 % probability when the true difference is 12 % (4 responses) and this probability rises as the true difference increases.

The final decision on the schedule for further investigation will be made by the Trial Steering Committee (TSC) in conjunction with the Trial Management Group (TMG), after considering the difference in complete remission rates, safety and tolerability, costs and MRD levels.

### Recruitment and consent

Participants will be recruited from approximately 18 research centres within the UK under the guidance of the NCRI (National Cancer Research Institute) CLL sub-group. The recruitment target requires 82 participants to be recruited into the trial, 41 in each treatment group. The trial opened to recruitment in October 2012 and is due to close in April 2016.

Patients will be approached during standard clinic visits for management of CLL and provided with verbal and written details about the trial. Informed consent will be collected by principal investigators at the participating hospitals. Participants will also be approached for the UK CLL Trials Biobank as discussed in the subsequent section on sub-studies.

### Randomisation

**Fig. 1:**
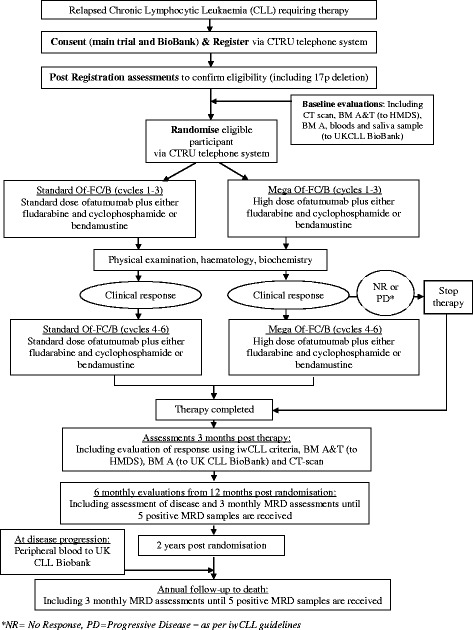
Trial flow diagram

Following confirmation of eligibility and consent, participants will be randomised into the trial by an authorised member of staff at the trial research site (Fig. 1). Randomisation will be performed centrally using the Leeds Clinical Trials Research Unit (CTRU) automated 24-hour telephone registration and randomisation system. Participants will be randomised on a 1:1 basis to receive either Standard Of-FC/B or Mega Of-FC/B. The choice of FC or B will be chosen prior to randomisation by the treating clinician or the participant. Participants are informed of their treatment allocation in this open study.

A computer-generated minimisation program that incorporates a random element will be used to ensure treatment groups are well balanced for the following participant characteristics: previous therapy containing a purine analogue such as fludarabine or bendamustine (Yes or No); age (≤65 or >65); gender; duration of previous remission (6–24 months or >24 months); choice of chemotherapy (FC or B).

### Protecting against bias

This trial cannot be blinded due to the different schedules of administration of the doses of ofatumumab. Treating consultants need to be fully aware of the drugs being administered to be able to treat reactions to treatment accordingly. The trial will use an independent randomisation service, and analyses will be on an intention-to-treat basis. The trial is administered by the Chief and Principal Investigators and the CTRU in accordance with Good Clinical Practice (GCP) guidelines. The primary endpoint response to treatment will be blindly assessed by an independent panel. Response will be assessed using the standard iwCLL criteria [9]. The assessment of response in the bone marrow for MRD will be centralised in a single laboratory, therefore providing standardisation for all assessments. So that the backbone chemotherapy will not be influenced by the outcome of the randomisation, it will be recorded on the randomisation system prior to the determination of the treatment allocation.

### Baseline assessments

Assessments to be performed prior to the participant starting treatment are a complete physical examination, WHO performance status, serology for hepatitis B and C, local haematology, biochemistry, a pregnancy test and an assessment of disease using blood, bone marrow aspirate, trephine and computerised tomographic (CT) scan. See the schedule of enrolment, interventions and assessments in Table 1.

### Intervention

**Fig. 2:**
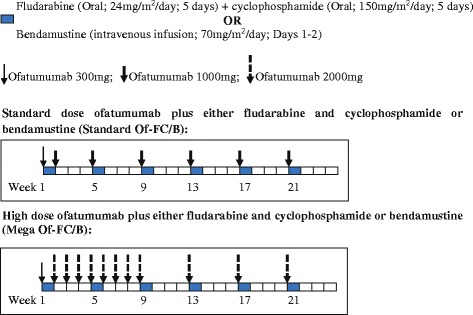
Diagrammatic representation of treatment schedule

Participants randomised to Standard Of-FC/B will receive 300 mg of ofatumumab intravenously (IV) on day 1 of cycle 1 and 1000 mg on day 8 of cycle 1 (Fig. 2). For cycles 2 to 6, they will receive 1000 mg of ofatumumab (IV) on day 1. Participants randomised to Mega Of-FC/B will receive 300 mg of ofatumumab (IV) on day 1 of cycle 1, followed by 2000 mg weekly for 8 doses, then 2000 mg 4-weekly for 3 doses. Both treatment groups will also receive backbone chemotherapy (FC/B).

Those who will receive FC will receive 24 mg/m^2^/day of fludarabine (orally) and 150 mg/m^2^/day of cyclophosphamide (orally) on days 1 to 5 for cycles 1 to 6. Cycles of FC are repeated every 28 days for a total of 6 cycles. Compliance with oral administration of FC will be recorded in the participant diary card for both trial arms. Those who will receive B will receive 70 mg/ m^2^/day on days 1 to 2 of cycles 1 to 6. Cycles of B are also repeated every 28 days for a total of 6 cycles. If nausea, vomiting or diarrhoea occurs with the prior cycle of therapy, FC should be given via the intravenous route from the next cycle onwards, due to concerns over drug absorption. The recommended IV schedule is 25 mg/m^2^/day of fludarabine for 3 days and 250 mg/m^2^/day of cyclophosphamide for 3 days.

Participants are assessed for their suitability for treatment within 1 week before each cycle according to standard clinical practice. As a minimum, tests will be performed to ensure dose reductions are implemented and concomitant medications are administered as per protocol.

The pre-medications are as follows. An analgesic (equivalent to 1000 mg of paracetamol) and antihistamine (equivalent to 10 mg of cetirizine) are administered prior to all ofatumumab infusions. Corticosteroid infusion (equivalent to 100 mg of prednisolone) is given for ofatumumab infusions 1 and 2, and for subsequent ofatumumab infusions a decreased corticosteroid (equivalent to 0–100 mg of prednisolone) may be given. The corticosteroid must be given within 30 minutes to 2 hours prior to each ofatumumab infusion. If the second infusion has been completed without the subject experiencing any Common Terminology Criteria for Adverse Events (CTCAE) grade 3 adverse events (AEs), pre-medication with corticosteroid may be reduced or omitted before the third to final ofatumumab infusions at the discretion of the investigator. If an infusion reaction occurs, the infusion should be temporarily slowed or interrupted.

The initial rate of the first infusions of 300 mg, 1000 mg or 2000 mg ofatumumab should be 12 mL/h, and if no infusion reactions occur, the infusion rate should be increased every 30 minutes to a maximum of 200 mL/h. For subsequent infusions of 1000 mg/2000 mg ofatumumab, if the previous infusion was completed without grade ≥3 infusion-associated AEs, then the infusion rate can start at 25 mL/h and should be doubled every 30 minutes to a maximum of 400 mL/h.

All participants will receive the following concomitant therapy: co-trimoxazole and acyclovir, to be commenced prior to therapy and continued until CD4+ counts are ≥200 cells/μL. Participants should receive 300 mg of allopurinol once daily, and should be prescribed anti-emetics as per local policy. No concomitant cytotoxic agents or radiotherapy should be given.

### Follow-up

A formal assessment of disease response will be made 3 months after the end of therapy, requiring the collection of blood, bone marrow aspirate, trephine and a CT scan as shown in Table 1. In order for the primary endpoint assessment of response to be as robust as possible and to ensure consistency and accuracy of reporting, the TMG have selected four CLL clinicians to make independent assessments of response (following the iwCLL criteria [9]), using anonymised data provided for each participant. Assessors will be blinded to treatment allocation and will not evaluate reports for participants treated at their centre. Each response assessment is made by two independent clinicians, and where the outcomes of the assessments differ, the data is sent to a third clinician, an independent arbiter, to make a final decision on response.

Follow-up visits to assess survival and MRD will be made at 12, 18 and 24 months post randomisation or until disease progression requiring therapy, if this is before 24 months post randomisation. Participants will also be assessed on a 3-monthly basis to monitor MRD status, which should continue until five consecutive MRD positive results have occurred. All participants will be followed up annually for survival until death.

All trial MRD testing will be carried out centrally by the Haematological Malignancy Diagnostic Service (HMDS) at Leeds Teaching Hospitals NHS Trust using the harmonised European Research Initiative on CLL (ERIC) method [13] to ensure standardisation of reporting.

### Safety reporting

Adverse reactions (ARs) are any untoward and unintended responses that are related to any dose administered of any trial treatment. These can be defined as any unintentional, unfavourable clinical sign or symptom; any new illness or disease, or the deterioration of existing disease or illness; or any clinically relevant deterioration in any laboratory assessments or clinical tests. ARs will be recorded from the start of treatment until 30 days after the last dose of trial treatment for all participants and will be evaluated for duration, intensity and causal relationship with the trial medication according to the National Cancer Institute Common Terminology Criteria for Adverse Events V4.0 [14] (NCI-CTCAE).

Serious adverse events (SAEs) and serious adverse reactions (SARs) are events that are fatal or life-threatening; require or prolong hospitalisation; are significantly or permanently disabling or incapacitating; constitute a congenital anomaly or a birth defect; or jeopardise the participant and require medical or surgical intervention to prevent one of the outcomes listed above. SARs are SAEs that are deemed to be possibly related to any dose administered of any trial treatment. Suspected unexpected serious adverse reactions (SUSARs) are SARs which are not listed in the reference safety information for that medicinal product. SAEs will be determined and recorded from the start of treatment until 30 days after the last dose of treatment. SARs and SUSARs should be reported from the start of treatment and for the duration of the trial.

An independent Data Monitoring and Ethics Committee (DMEC) will review the safety and ethics of the trial. The CTRU will prepare detailed unblinded reports for the DMEC at approximately yearly intervals. Unblinded safety updates are also prepared for review at 3-monthly intervals whilst participants are receiving trial treatment.

### Data collection

Data will be collected on paper case report forms and entered into a validated trial database by the CTRU. A validation check program will be incorporated into the trial database to verify the data, and discrepancy reports will be generated for resolution by the investigator. Priority validations will be incorporated into the validation programme to ensure that any discrepancies related to participant rights or safety are expedited to sites for resolution. Data will be monitored for quality and completeness by the CTRU. Missing data will be chased until received, and confirmed as not available or the trial is at analysis. The CTRU/sponsor will reserve the right to intermittently conduct source data verification exercises on a sample of participants, which will be carried out by staff from the CTRU/sponsor. Source data verification will involve direct access to participant notes at the participating hospital sites and the central collection of copies of consent forms and other relevant investigation reports. Data will be held on a secure server at the University of Leeds and paper case report forms stored in a locked unit, both of which are accessible only to authorised trial staff.

### Statistical methods and analysis

The CTRU statisticians will be responsible for the statistical analysis, and a final statistical analysis plan will be written before any analysis takes place.

All analyses will be conducted on the intention-to-treat (ITT) population, where participants will be included according to the treatment arm to which they are randomised as long as they receive at least one dose of trial treatment. Participants who are randomised but do not receive any trial treatments will be replaced, since this is a phase II trial. A per-protocol (PP) analysis will also be conducted for the primary endpoint if there are a considerable number of protocol violators. The safety population will consist of all participants who received at least one dose of trial treatment, where participants will be included according to the treatment they actually received.

The response endpoints (complete remission, overall response and MRD response) will be summarised by treatment group and 95 % confidence intervals reported. The number and proportion of participants in each individual response category will also be reported.

If there are less than 10 complete remissions out of the first 37 evaluable participants in each treatment arm, the treatment will be deemed unsuitable to take forward for further investigation. In the case where both treatment arms are acceptable, the schedule to be taken forward will be chosen based on the efficacy selection criteria (a difference of at least three complete remissions). Otherwise, the selection will be determined by the TMG in discussion with the DMEC and TSC based on all the available endpoint data and treatment information.

Survival analyses will be carried out for the endpoints progression-free survival, overall survival and time to MRD relapse in participants who are MRD negative when all randomised participants have been followed up for a minimum of 2 years from randomisation. Survival will be assessed using Cox regression, adjusting for the stratification factors, and summarised for each arm using Kaplan-Meier survival curves and reporting median survival times.

The MRD results over five time points once MRD is detectable (MRD positive) will be used to fit a model to the MRD data to assess the growth pattern of MRD. Individual growth dynamics are assumed to follow the exponential distribution, where the rate of increase of MRD levels is assumed to be constant over time. Individual growth curves will be fitted for all participants in order to obtain estimates of the intercept and growth parameters for each participant. Assuming independence between the parameters, they will be incorporated into a Cox regression analysis on progression-free survival (PFS) and overall survival (OS) to obtain estimates of the change in hazard for every unit increase in the growth parameters, in order to model the relationship between initial level of MRD once detected, MRD growth rate and survival.

Safety analyses will summarise all SUSARs, SARs, SAEs, ARs and treatment-related mortality rates as well as laboratory changes. Safety data will be presented by treatment group for the safety population in addition to relationship to study treatment.

Exploratory analyses may be carried out to assess whether the following are prognostic of the primary outcome: randomisation stratification factors; β_2_-microglobulin; Binet stage; immunoglobulin variable heavy chain gene (VH) mutation status; and 11q deletion, 13q deletion and trisomy 12 on FISH. These analyses may, by chance, generate false negative or positive results, and so those carried out will be interpreted with caution and treated as hypothesis-generating.

### Protocol amendments

The trial opened to recruitment in October 2012 using version 2 of the protocol. The protocol was amended to version 4 in April 2014 to include the additional chemotherapy backbone of bendamustine in response to changing clinical practice. This amendment was reviewed and approved by the sponsor, funder, DMEC, TSC and Medicines and Healthcare Products Regulatory Authority (MHRA) and REC. Protocol amendments are disseminated to relevant parties by the CTRU.

### Sub-studies

Trial participants are also invited to take part in the UK CLL Trials Biobank at the University of Liverpool. Participants are approached at baseline with a separate consent form (REC reference 14/NW/1014), and biological samples are sent to the University of Liverpool.

### Trial organisation and administration

The trial was developed by the COSMIC TMG, with the support of the UKCLL/NCRI CLL Clinical Trials Sub-Group. The trial is funded by GlaxoSmithKline (GSK) and endorsed by the Clinical Trials Awards and Advisory Committee (CTAAC) of Cancer Research UK. The trial is sponsored by the Leeds Teaching Hospitals NHS Trust (R&D Office, 34 Hyde Terrace, Leeds, LS2 9LN), run and co-ordinated by the CTRU, University of Leeds, and is registered (ISRCTN51382468, EudraCT Number 2011-000796-14). The trial will be conducted in accordance with the principles of Good Clinical Practice (GCP) in clinical trials, as applicable under UK regulations, the NHS Research Governance Framework and through adherence to CTRU Standard Operating Procedures (SOPs). The CTRU and the sponsor have systems in place to ensure that serious breaches of GCP of the trial protocol are identified and reported. Ethical approval has been obtained from the National Research Ethics Service Committee Yorkshire & The Humber – Leeds East (reference 11/YH/0260). No additional compensation for harm was provided for trial participants over that which is available to NHS patients. Ofatumumab was only provided for the duration of protocol treatment, and subsequent treatment is as per standard care.

A core project team, a TMG, a TSC and a DMEC have been established. The independent DMEC review the safety and ethics of the trial alongside trial progress, and the overall direction is overseen by the TSC. Three-monthly interim safety reports are presented to the DMEC with a full review annually. The DMEC, in light of the interim data and of any advice or evidence they wish to request, will advise the TSC if there are any concerns or reasons that the trial should not continue. The results of the study will be published in peer-reviewed journals and will be presented at relevant national and international conferences. There are no plans to use professional writers for presenting the outcomes of this trial. The CTRU will control the final trial dataset, and any requests for access will be reviewed by the TMG and TSC, subject to existing contractual arrangements with the funder. The protocol, sample case report forms and participant information are available on a case-by-case basis as agreed to by the TMG, upon request to the corresponding author. A SPIRIT checklist has been prepared for this manuscript.

## Discussion

At the time of design, there was limited evidence regarding the optimal treatment of patients with relapsed or refractory CLL, and suitable therapies were urgently needed. The COSMIC trial is designed to use pre-determined stopping rules to assess whether standard or high dose ofatumumab in combination with chemotherapy will be suitable for carrying out a further phase III trial.

Recruitment has been challenging for COSMIC. The original trial planned to recruit the required 82 participants by March 2014, and to achieve this target the recruitment period was extended to March 2016. Recruitment numbers provided during feasibility assessment were significantly higher than observed during trial recruitment. We believe this was predominantly due to alternative treatments for relapsed CLL becoming available within clinical trial settings. Ibrutinib and idelalisib both commenced evaluation within a clinical trials context during the lifetime of the trial and have become a more favourable treatment option to some patients. The TMG, TSC and UKCLL/NCRI CLL Clinical Trials Sub-Group regularly reviewed (at least annually) whether the trial was still asking a relevant clinical question.

The availability of chemotherapy within the NHS has posed an additional challenge to executing the trial. Bendamustine was made available for relapsed CLL through the Cancer Drugs Fund (CDF) in June 2013 and was added to the trial protocol in April 2014. This was carefully considered in terms of the trial design and its impact on the analysis plan, which was agreed upon with the oversight committees. The CDF then removed access to bendamustine in September 2015 for new patients, which impacted further on recruitment to the trial, showing the importance of the changing treatment environment during the lifetime of a trial.

The findings of the COSMIC trial aim to inform the design and development of a future definitive trial, which could enhance the treatment of patients with relapsed CLL who are refractory to chemotherapy.

### Trial status

The COSMIC trial opened to recruitment in October 2012 and is due to close to recruitment in April 2016.

## Additional file

Additional file 1: SPIRIT checklist. (DOC 122 kb)

## Abbreviations

AE: Adverse event
AR: Adverse reaction
B: Bendamustine
BMA/T: Bone marrow aspirate/trephine
CI: Confidence interval
CLL: Chronic lymphocytic leukaemia
CR: Complete remission
CR(i): Complete remission with incomplete marrow recovery
CRF: Case record form
CRUK: Cancer Research UK
CTAAC: Clinical Trials Awards and Advisory Committee (CRUK)
CTRU: Clinical Trials Research Unit, University of Leeds
DMEC: Data Monitoring and Ethics Committee
EMA: European Medicines Agency
FC: Fludarabine and cyclophosphamide
FCM: Fludarabine, cyclophosphamide and mitoxantrone
FCM-R: Fludarabine, cyclophosphamide, mitoxantrone and rituximab
FCR: Fludarabine, cyclophosphamide and rituximab
FDA: Food and Drug Administration
FISH: Fluorescence in situ hybridisation
GCP: Good Clinical Practice
HIV: Human immunodeficiency virus
HMDS: Haematological Malignancy Diagnostic Service
ITT: Intention to treat
IV: Intravenous(ly)
iwCLL: International Workshop on Chronic Lymphocytic Leukaemia
MRD: Minimal residual disease
NCRI: National Cancer Research Institute
NHS: National Health Service
NICE: National Institute for Health and Care Excellence
Of-B: Ofatumumab with bendamustine
Of-FC: Ofatumumab with fludarabine and cyclophosphamide
OS: Overall survival
PFS: Progression-free survival
PS: Performance status
REC: Research Ethics Committee
SAE: Serious adverse event
SOP: Standard Operating Procedure
SPIRIT: Standard Protocol Items: Recommendations for Interventional Trials
SUSAR: Suspected unexpected serious adverse reaction
TMG: Trial Management Group
TSC: Trial Steering Committee
WHO: World Health Organisation
